# Green Urine

**DOI:** 10.1100/tsw.2011.101

**Published:** 2011-05-26

**Authors:** Diptesh Gupta, Raghav Gupta

**Affiliations:** ^1^ Department of Internal Medicine, University of Missouri – Columbia, USA; ^2^ Department of Internal Medicine, St. Luke's Hospital, St. Louis, MO, USA

**Keywords:** green, urine, propofol

## Abstract

Urinalysis is an integral part of a thorough patient evaluation. Change in urine characteristics can give clues to help solve some of the diagnostic challenges faced by physicians. We discuss a case of a benign cause of green discoloration of urine caused by propofol infusion, which reversed following its discontinuation.

Discoloration of the urine may indicate the presence of an underlying systemic disorder. However, it can also be due to ingested substances or drugs, or related to the Foley catheter[1,2,3]. This case is of a 64-year-old male who developed green-colored urine secondary to propofol administration for the purpose of sedation in the intensive care unit. Propofol is a commonly used intravenous drug for sedation in the operating room or intensive care. The green urine color due to propofol is thought to be a result of its phenolic green metabolite produced in the liver and excreted in the urine[2,4]. This green discoloration associated with propofol is benign, non-nephrotoxic, and reversible with drug discontinuation[1,2]. Interestingly, propofol has also been reported to cause green discoloration of the hair and liver, and, on occasion, a transient white or pink urine color; the latter thought to be a result of uric acid crystal precipitation[1]. These side effects of propofol are seen with a continuous infusion, but rarely seen in patients during induction of anesthesia. Green urine discoloration is not unique to propofol. Other potential causes include[1]:

1. Drugs such as cimetidine, promethazine, amitriptyline, indomethacin, metoclopramide, and flutamide
2. Ingestion of substances such as methylene blue and indigo blue dyes in enteral feedings, or asparagus
3. Metabolic disorders such as Hartnup disease
4. Urinary tract infection with *Pseudomonas*, which can lead to accumulation of pyocyanin pigment
5. Structural abnormalities such as vesicoenteral fistulas that cause excretion of bile into the urine

A thorough review of medications, nutritional supplements, and urine tests focusing on the presence of biliverdin or infection should help to identify the cause of urine discoloration in most patients.
